# AI Revolution in Upper GI: Endoscopic Diagnosis of Gastric Cancers and Premalignant Neoplasms

**DOI:** 10.3390/diseases14090333

**Published:** 2026-09-12

**Authors:** Ivan Shun Fai Lau, Hon Chi Yip, Philip Wai Yan Chiu, Martin Chi Sang Wong, Louis Ho Shing Lau

**Affiliations:** 1 Department of Medicine and Therapeutics, The Chinese University of Hong Kong, Hong Kong; sfilau@cuhk.edu.hk; 2 Institute of Digestive Disease, The Chinese University of Hong Kong, Hong Kong; hcyip@surgery.cuhk.edu.hk (H.C.Y.); philipchiu@cuhk.edu.hk (P.W.Y.C.); 3 Department of Surgery, The Chinese University of Hong Kong, Hong Kong; 4 The Jockey Club School of Public Health and Primary Care, The Chinese University of Hong Kong, Hong Kong; wong_martin@cuhk.edu.hk; 5 Li Ka Shing Institute of Health Sciences, The Chinese University of Hong Kong, Hong Kong

**Keywords:** AI, artificial intelligence, gastric cancer, CAQ, CADe, CADx, esophagogastroduodenoscopy, endoscopy, intestinal metaplasia, EGGIM

## Abstract

**Background**: Discrepancies between endoscopic and pathological diagnoses commonly occur. From pre-malignant neoplasms to early gastric cancers (EGC), this inconsistency often leads to significant alteration of management. AI (artificial intelligence) assisted diagnostic tools have the potential to mitigate the differences. From optimizing endoscopic examination through standardizing quality and risk stratification by identifying at-risk populations to enhancing EGC detection and characterization guiding therapeutic interventions, AI-assisted endoscopy has shown potential to overcome current limitations in standard care. **Methods**: In this state-of-the-art narrative review, we synthesize the current evidence and prevailing paradigm, discuss existing limitations, and outline the future directions of AI-assisted endoscopy in EGC. **Results**: AI models demonstrate potential in enhancing all steps of the EGC management pathway, from optimizing endoscopy quality, identification of at-risk populations, and lesion detection, lesion characterization, to endoscopic resection guidance and lymph node metastasis prediction. Yet, existing evidence largely remains retrospective, with interventional data demonstrating heterogeneity. The conflicting data largely stems from inherent fundamental limitations of AI research in EGC, such as the lack of standardized objective performance metrics, inconsistent methodologies, and reporting standards. These issues fundamentally hinder generalizability and implementation in routine clinical practice. **Conclusions**: AI-enhanced endoscopy demonstrates potential to overcome current limitations in EGC management. Despite this promising future, there are obstacles to validation, implementation, and mitigating disparities owing to inherent fundamental issues.

## 1. Introduction

Gastric cancer remains one of the most prevalent malignancies globally with significant associated morbidities and mortalities [1]. Despite improvements in key domains such as diagnostic capabilities, *H. Pylori* eradication, and curative resection techniques, the discrepancy between endoscopic and final histopathological diagnosis remains a key limiting step [2,3], spearheading substantial gastric cancer-related morbidities. This problem is pertinent in all aspects of gastric cancer care. However, it is the most critical in the management of EGC.

**Figure 1:**
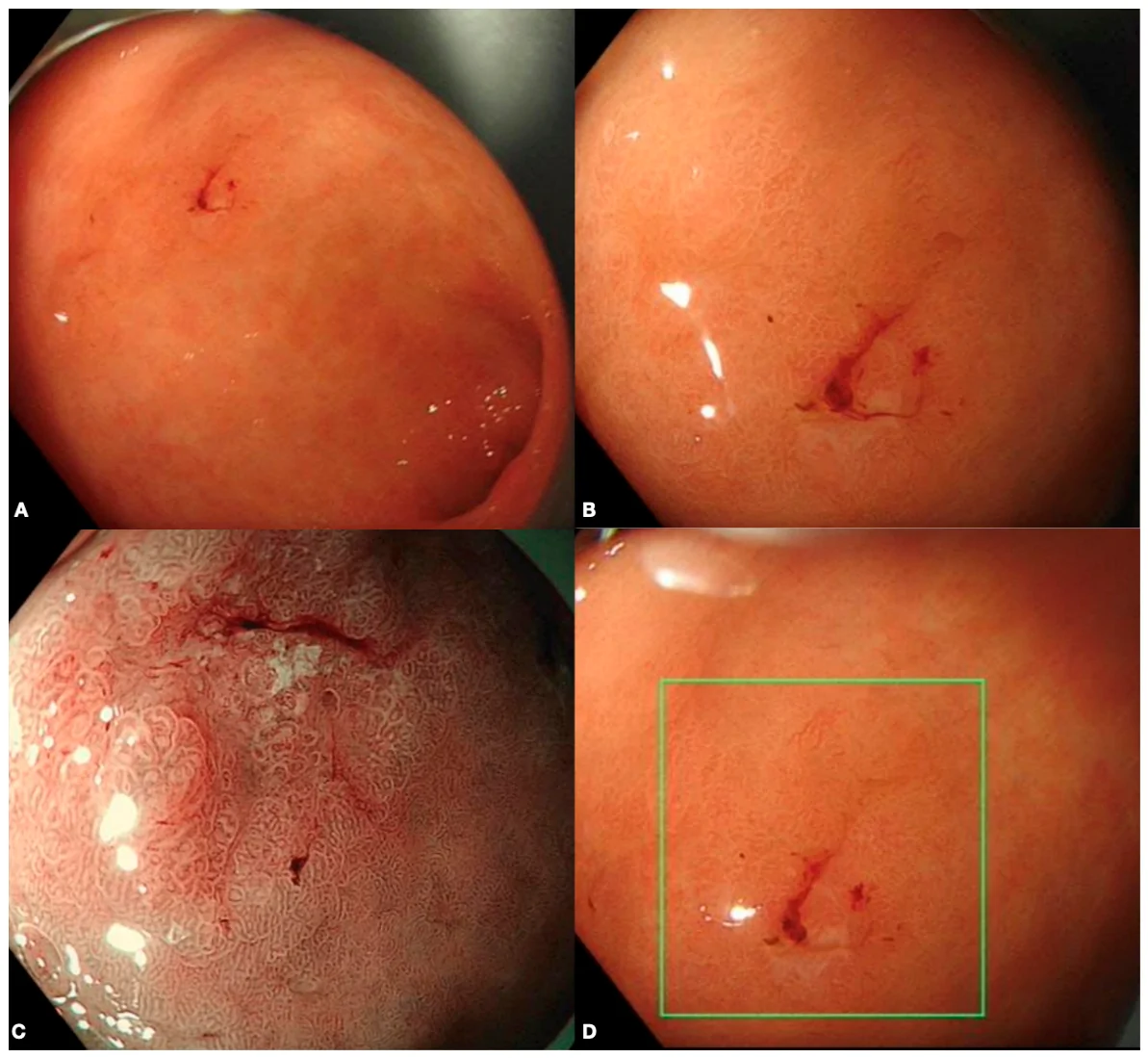
An Early Gastric Cancer (EGC) in Different Image Modalities. (**A**) White Light Endoscopy (WLE) examination of gastric antrum. (**B**) Focused WLE examination of EGC. (**C**) Narrow Band Image (NBI) examination of EGC. (**D**) AI-assisted detection of EGC with OIP-Ge1, Olympus Corporation, Japan. Image courtesy of the authors.

The paradigm shift from traditional surgical resection towards curative endoscopic resection has substantially improved patient-level outcomes for EGC, whilst mitigating the medical burden historically associated with more invasive approaches [4]. Nonetheless, these improvements in clinical outcomes are counterbalanced by challenges in case identification, with suboptimal concordance rates in EGC diagnosis limiting reliable patient selection [5]. The treatment pathway greatly differs between EGC, which is confined to the submucosal layer, and advanced gastric cancer which extends beyond the submucosal layer with potential lymphovascular invasion (LVI). This distinction alters a local endoscopic resection to gastrectomy with lymph node resection, subsequently affecting survival rates and quality of life [6,7]. Identification and diagnosis of EGC is dependent on subtle differences in endoscopic features such as mucosal color changes, protrusion or depression of the lesion commonly seen under white light endoscopy (WLE) examination, or abnormal microvascular and micro-surface patterns under enhanced narrow band imaging (NBI) endoscopy (Figure 1) [8]. Ultimately, this predicts key pathological features, for example, histological differentiation type, invasion depth, and lymphovascular invasion [8]. This determines whether curative resection can be successful for EGC [4]. The subsequent clinical decision-making process hinges upon the concept of endoscopic resectability, which is guided by the estimated risk of lymph node metastasis (LNM). The indications for endoscopic resection are well-established for lesions with a negligible (<1%) risk of LNM, typically comprising differentiated-type intramucosal cancers ≤2 cm in size without ulceration [9]. These indications continue to become more inclusive, supported by accumulating clinical evidence, which now include selected lesions such as differentiated-type mucosal cancers >2 cm without ulceration and, more recently, selected undifferentiated-type mucosal cancers ≤2 cm without ulceration [10]. These criteria, primarily derived from the Japanese Gastric Cancer Treatment Guidelines and endorsed by European Guidelines [11], form the foundation for determining whether a lesion is suitable for curative endoscopic resection. Owing to the subtle features and variable expertise, endoscopic diagnosis is hindered by suboptimal detection ability and concordance rates, both between endoscopists and between endoscopic diagnosis and final histopathological result [5]. This problem is compounded by variability in endoscopic quality, stemming from similar limitations in expertise and time-resource constraints. Likewise, similar diagnostic discrepancies occur during risk stratification and identification of patients at highest risk of EGC. Gastric intestinal metaplasia (GIM) is a stepwise precursor to EGC, which is prevalent particularly in Asians [12]. Populations with severe GIM are at significant risk of developing or harboring EGC, hence should receive interval screening according to established international guidelines [11]. Classically, risk stratification tools predominantly required histological assessment of GIM severity and subsequent delineation of risk. Currently, risk stratification tools such as Endoscopic Grading of Gastric Intestinal Metaplasia (EGGIM) have been shown to have high concordance rates with histopathological equivalent tools, identifying those at risk of EGC reliably and becoming increasingly adopted worldwide [13]. Performed under NBI, color and mucosal differences are detected, then rated for extensiveness in specified areas. Subsequently, a combined score is calculated to risk stratify using EGGIM [13]. However, it requires specific endoscopic expertise, limiting reproducibility beyond trained users. AI-assisted endoscopy has the potential to mitigate the discrepancy between endoscopists, minimizing the demand required to accurately and reliably identify high-risk individuals and diagnose EGC consistently.

Beyond detection, AI-assisted endoscopy has also shown promise in the local staging of EGC, enhancing the accuracy of case selection and guiding therapeutic decision-making to maximize the potential of curative endoscopic resection. Latest AI models in endoscopy have applied Convolutional Neural Networks (CNNs), a deep learning (DL) architecture inspired by the organization of the visual cortex, designed to learn features automatically and adaptively from provided images. By processing endoscopic images through successive layers that detect edges, shapes, and increasingly complex patterns, CNNs can be trained to recognize subtle mucosal and vascular changes imperceptible to the human eye. Ultimately, these models can generate predictions that match or exceed human performance in specific diagnostic tasks. It is this capacity for automated, reproducible pattern recognition that positions CNN-based systems as powerful tools to address the persistent discrepancies in endoscopic examinations [14].

This state-of-the-art narrative review aims to present the latest developments in AI-assisted endoscopy in EGC. We begin with AI-assisted quality control of endoscopy, followed by the identification of at-risk populations, the detection of EGC, and finally the characterization and local staging of lesions. Lastly, we evaluate obstacles to validation and implementation of AI-assisted endoscopy, illustrating the inherent fundamental limitation of AI systems in EGC.

## 2. Methods

The narrative review approach was selected to permit a broader synthesis of the literature, better suited to contextualizing current paradigms and discussion of future directions for AI in EGC endoscopy.

**Figure 2:**
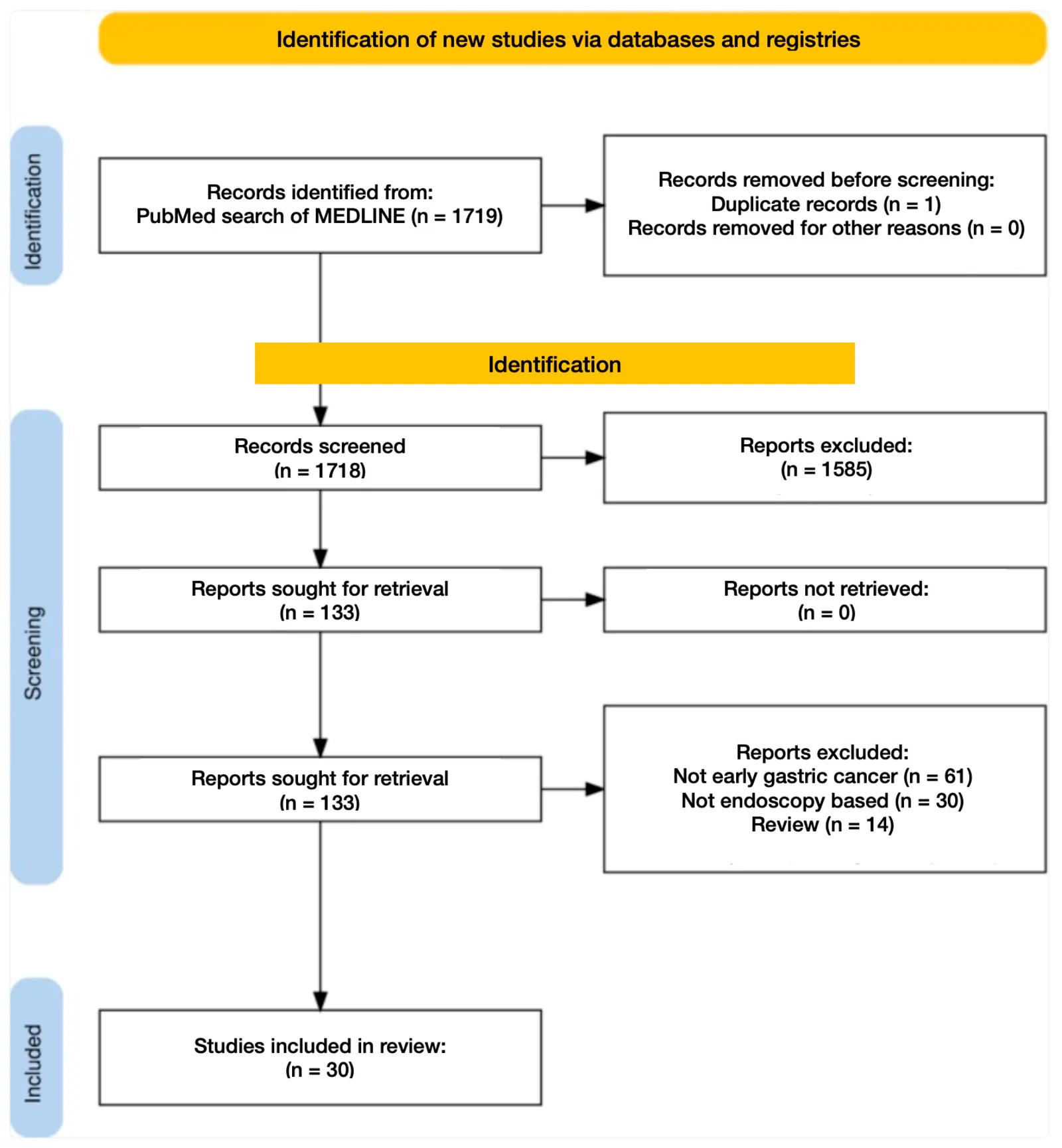
Literature search process with results from PubMed demonstrated.

A comprehensive literature search was conducted across PubMed, Embase, and Medline databases from inception through 30 June 2026. Search terms included the following groups of keywords: AI-related terms (e.g., artificial intelligence and machine learning), endoscopy-related terms (e.g., endoscopy and gastroscopy), and early gastric cancer plus GIM-related terms (e.g., gastric neoplasm, early gastric cancer, and gastric intestinal metaplasia) combined using Boolean operators “AND” and “OR.” To identify additional relevant publications, the reference lists of retrieved studies were manually screened. Only studies published in English were included. The search workflow is illustrated in Figure 2.

## 3. Results

### 3.1. Image Preprocessing and Its Implications Across Imaging Modalities

Image preprocessing is a fundamental step in the development of imaging AI models, which often influences diagnostic performance and generalizability. While systematic reviews dedicated exclusively to preprocessing methodologies for EGC are limited, the existing literature highlights several established techniques employed across studies.

CNN is the technique applied in the latest paradigm of AI in EGC; hence, we will briefly discuss the image preprocessing methods used in this technique. The typical pipeline begins with image standardization, including resizing, color normalization, and de-noising, and often includes removal of sub-optimal images. The aforementioned is commonly done manually and remains a core principle regardless of techniques applied to generate AI models. The importance of these preprocessing decisions is underscored by a systematic review and meta-analysis on deep learning for detecting EGC, which identified significant heterogeneity between imaging modality and training dataset characteristics across studies, with models trained on larger and more diverse datasets demonstrating more robust performance [15].

Data augmentation is critical to CNN training, often applied to artificially expand the training set and prevent overfitting. Techniques such as rotation, flipping, scaling and brightness or contrast adjustments are common practice. To address the challenge of limited medical datasets, more sophisticated augmentation methods have been developed. Techniques such as Poisson Blending are advanced image processing techniques used to insert a source image patch into a target background without visible seams, allowing realistic synthesis of abnormal lesions onto normal mucosal images [16]. Another approach combines multiple methods, such as the use of AutoAugment’s ImageNet policy in combination with cut-and-paste techniques using a sliding window algorithm. This involves learning optimal augmentation strategies from large-scale datasets and strategically overlaying lesion patches onto normal images in a systematic manner to expand training samples. These methods have demonstrated meaningful improvements in classification performance compared with models trained on original datasets alone [16,17].

Beyond augmentation, CNN models often employ techniques to highlight suspicious regions. Gradient-weighted Class Activation Mapping (Grad-CAM) is a well-established technique that generates heatmaps, which are color-coded overlays visually indicating the most influential region of an image in the model’s diagnostic decision. This allows the decision-making process to be more transparent and interpretable, ultimately enhancing clinical trust [18].

The choice of preprocessing is often adapted to leverage the strengths of each imaging modality. NBI enhances mucosal and vascular visualization through optical filtering; hence, preprocessing aims to emphasize the enhancement of these features. In contrast, preprocessing for WLE is more commonly focused on standardization and artifact removal, correcting for inconsistencies and aiming to create a more uniform dataset for model training. As NBI may not be universally available, an advanced preprocessing strategy involves cross-modal image synthesis. A recent retrospective study by Lei et al. demonstrated that WLE images can be converted into virtual NBIs using Stable Diffusion effectively [19]. However, while virtual NBI has shown diagnostic accuracy comparable to traditional NBI under human examination, its efficacy when integrated into AI models, in particular for CNN-based classification, has yet to be established.

Despite these advancements, considerable variability in preprocessing pipelines exists across EGC AI studies. Differences in patch size, augmentation strategies, and preprocessing methods hinder standardization and challenge model generalizability. While some studies detail their preprocessing comprehensively, others provide only limited descriptions. This underscores the need for consensus recommendations to ensure methodological transparency and reproducibility in future EGC AI endoscopy research.

### 3.2. Enhancing Endoscopic Quality and Risk Stratification with AI

#### 3.2.1. Optimizing Quality, Standardizing Practice: AI in Endoscopic Quality Assurance (CAQ)

Endoscopic examination quality is directly correlated with EGC detection rates [20]. However, maintaining consistent quality is difficult owing to time–resource limitations and variable expertise. AI-assisted quality control aims to homogenize practice and enhance quality by ensuring compliance with key quality metrics, including minimization of blind spots, systematic landmark identification, and automated photo documentation [21,22,23,24]. Notably, multiple interventional studies have illustrated the ability to enhance gastric neoplasm detection rate through computer-assisted quality control [21,24].

A recent multicenter randomized controlled trial (RCT) by Chen S et al. demonstrated an increased upper GI neoplasm detection rate with the use of AI-assisted endoscopic quality control [21]. Among over 32,000 randomized patients, the AI-assisted arm showed a relative risk improvement of 1.527 (95% CI: 1.387–1.681, *p* < 0.001) for gastric cancer detection specifically. An operation score determining the completeness and quality of endoscopy, also showed improvement. However, there was a 22% increase in examination time, from 5.60 to 6.84 min (*p* < 0.001). While the findings underscore the potential of computer-assisted quality control in real-world settings, it remains difficult to disentangle whether the observed improvements are attributable to the AI system itself or largely driven by the extended examination time. Adequate endoscopic examination time has been shown to improve detection rates of gastric neoplasms [25]. Additionally, the study reported a positive correlation between procedure duration, quality metrics, and lesion detection. Furthermore, the research environment itself and the potential for the AI program to modify endoscopist behavior towards more thorough examination may have contributed to the enhanced outcomes. Thus, further research is required to elucidate the complex, multifactorial relationship between AI-assisted quality control, examination time, and detection efficacy.

#### 3.2.2. Stratifying Risk, Standardizing Care: AI in Endoscopic Risk Assessment of EGC

Identification and selection of the population at highest risk of EGC is critical to optimizing sensitivity of any EGC diagnostic tool. Traditionally, GIM has been risk-stratified through histological grading alone. Lately, the Endoscopic Grading of Gastric Intestinal Metaplasia (EGGIM) classification, endorsed by multi-society guidelines for its ability to stratify risk using endoscopic images alone, is increasingly adopted internationally [11]. It has been shown to have high concordance rates with histopathological assessment tools [13]. The EGGIM is a total score examining and combining five distinct areas of the stomach under NBI assessment. A combined score of >4 indicates extensive intestinal metaplasia, necessitating interval endoscopic surveillance for gastric cancer [13]. This reliance on sole visual assessment makes it a particularly suitable candidate for augmentation by AI. The development of AI-assisted endoscopic systems is specifically motivated by the need to overcome the inherent limitations of human visual evaluation, particularly the demands of specialized training and the potential for inter- and intra-observer variability. Hence, AI facilitates EGGIM scoring to become more objective and replicable for stratification of those at highest risk of EGC. A recent study by Almeida et al. demonstrates CNN can automate the use of EGGIM, identifying patients requiring active surveillance for EGC with 100% sensitivity and negative predictive value. Specifically, the CNN showed high accuracy and sensitivity for EGGIM grading of individual images compared to experts’ evaluation and the correct risk stratification of high-risk patients with the EGGIM score [26].

This is the first study utilizing AI in endoscopy to risk stratify precancerous neoplasms in the stomach. With high accuracy, sensitivity and negative predictive value, the study demonstrates potential in the identification of populations at highest risk of EGC. Although specificity was comparatively lower than other AI tools in detecting GIM [27], its value is in the ability to grade and formulate total per-patient scores consistently. This first-in-class tool encompasses both identification and severity assessment, ultimately allowing accurate risk stratification and enhancing clinical decision-making. Despite these promising results, several limitations impact its generalizability: (1) The study is a single-center study with 65 fully documented patients. (2) The tool required optimization and high-quality images. In particular, it required the use of Olympus endoscopes by expert endoscopists with >5 years of system experience. Additionally, suboptimal frames (e.g., those containing food, foam, or motion blur) were excluded. This suggests its performance in less controlled real-world settings remains to be demonstrated. Furthermore, the experiment was conducted on selected still images with manually cropped patches, rather than real-time video images, which deviates from real-world practice. The dynamic environment captured by real-time video, which includes assessment of structures in three dimensions and features relative to surroundings such as color, size and texture, is a crucial element for thorough evaluation. While it could be argued that image optimization and still-frame assessment are inherent prerequisites for endoscopic evaluation, the transition into real-time video analysis, supported by robust multicenter prospective validation, would undoubtedly accelerate the integration of AI-assisted EGGIM into routine clinical workflows and solidify its definitive role in risk stratification.

### 3.3. Diagnosing the Undetectable: Computer-Assisted Detection and Diagnosis of EGC

**Figure 3:**
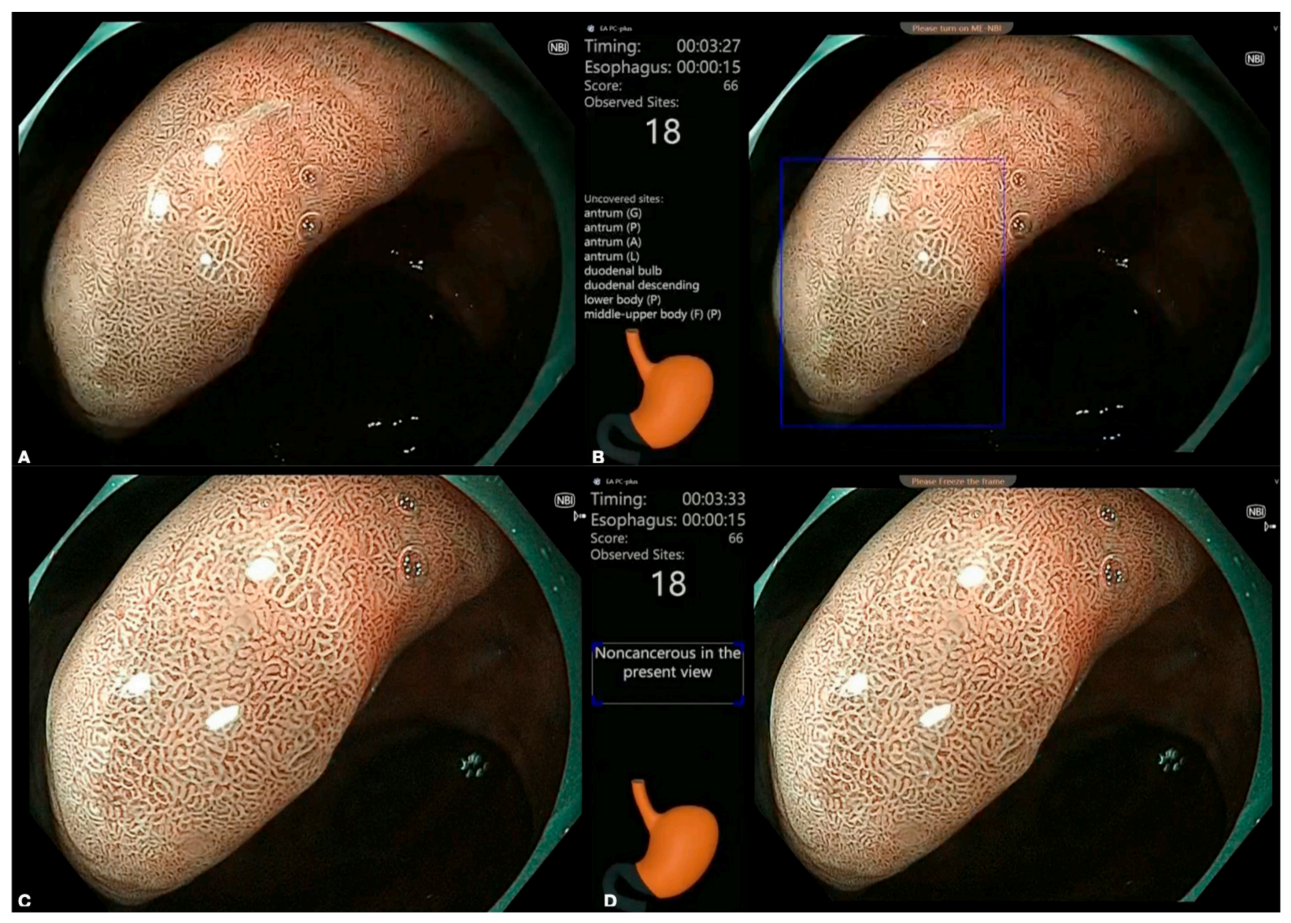
Multi-functional artificial intelligence platform in endoscopic diagnosis of EGC (EndoAngel UGI, Wuhan, China)**.** (**A**) NBI examination without AI assistance. (**B**) EndoAngel computer-aided detection, with the lesion highlighted in a blue box. Computer-aided quality assurance with real-time monitoring of blind spots in the stomach, highlighted in orange. (**C**) NBI examination with near-focus inspection, without AI assistance. (**D**) EndoAngel computer-aided diagnosis, with predicted histopathology located in the white box. Image courtesy of the authors.

**Figure 4:**
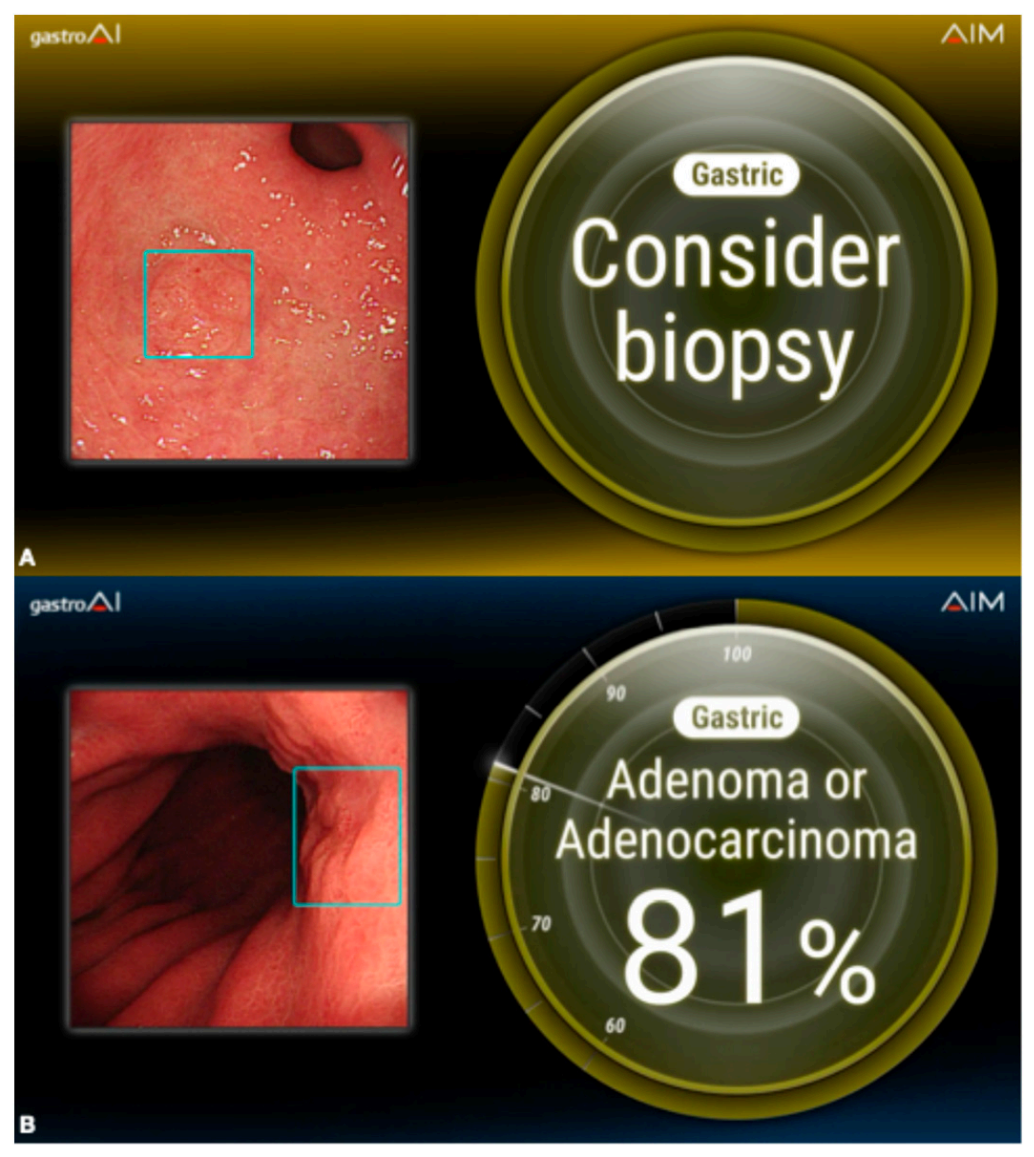
Endoscopic image diagnosis support software—gastroAI-model G (AI Medical Service Inc., Tokyo, Japan) (**A**) highlights suspicious area for biopsy and (**B**) predicts the probability of advanced pathologies. Image courtesy of AI Medical Service Inc. (Japan).

The detection and diagnosis of EGC is often hindered by a combination of factors, including subtle endoscopic features, variable operator expertise, and time limitations [28]. Identification of minute mucosal changes is not only time-consuming but also demands extensive training to achieve proficiency. In routine clinical practice, WLE remains the most used modality for initial mucosal screening, including the assessment of non-malignant pathologies. When a suspicious neoplastic lesion arises, WLE is frequently complemented with NBI to better delineate surface and vascular patterns, characterizing the underlying pathology for diagnosis. AI in endoscopy seeks to enhance lesion detection and reduce diagnostic variability (Figure 3 and Figure 4). Since the advent of machine learning (ML), numerous studies have investigated the application of AI to both WLE and NBI in the context of EGC detection and diagnosis [15,18]. The latest paradigm involves DL methods, predominantly CNNs. As each AI model is developed for a specific indication, it is essential to understand their performance across different assessment modalities and clinical contexts. This diversity is reflected in the current literature, where numerous systematic reviews address distinct subspecialty areas and a notable shift toward prospective clinical trials, underscoring the need for a structured synthesis to further understand the current paradigm (Table 1). In this section, we present the latest systematic reviews on Computer-Aided Detection (CADe) of EGC in WLE, comparisons between WLE and NBI for Computer-Aided Diagnosis (CADx) of EGC, its impact on less experienced endoscopists, and prospective interventional studies that evaluate real-world effectiveness. The below reported comparative performance between AI and endoscopists is based on image-only interpretation, unless otherwise specified.

#### 3.3.1. CADe: WLE for EGC

A recent systematic review and meta-analysis by Liu and colleagues offers a comprehensive evaluation of ML’s role in detecting EGC under WLE. Their findings confirm that ML algorithms can achieve a level of accuracy on par with experienced endoscopists [15]. A total of 583 unique studies published up to July 2025 were screened. A total of 15 studies met the strict predefined criteria, which required pathologically confirmed EGC, exclusive use of WLE and ML models, and the reporting of sensitivity and specificity as primary outcomes. All ML models in this study were CNN-based. Encompassing over 37,000 images, a bivariate random-effects model pooled diagnostic data from internal validation sets across all 15 studies. The pooled analysis demonstrated high sensitivity and specificity, reaching 0.91 (95% CI: 0.82–0.95) and 0.93 (95% CI: 0.87–0.97), respectively. However, when only the 4 studies of external validation from an independent dataset totaling 3579 images were considered, performance dropped with a pooled sensitivity of 0.82 (95% CI: 0.61–0.93) and specificity of 0.83 (95% CI: 0.74–0.90). Although there was a performance drop-off when compared to expert endoscopists, further analysis revealed no statistically significant difference in diagnostic performance between the two groups [15], underscoring the potential of these algorithms to match expert-level clinical acumen.

This meta-analysis highlights the promise of CNN models applied to WLE as a clinical decision-support tool. Such technology could help mitigate the well-documented inter-rater variability, in which a significant portion stems from variability of endoscopic experience, achieving a detection level comparable to experts. However, generalizability of the meta-analysis is limited. The cohort comprises largely retrospective and single-center designs. In addition, only two studies were trained on large-scale public datasets, with the remainder utilizing smaller, institutional image datasets. This partially explains the notable performance decrement observed between internal and external validation. Lastly, the performance metrics in this meta-analysis were derived from curated, often high-quality, static image datasets. Such idealized conditions may not fully reflect the effectiveness of these models when deployed in real-time during the workflow of a busy endoscopy unit. This distinction is particularly pertinent given the inherent nature of WLE, where mucosal evaluation is dynamic, under time constraint and can be compromised by luminal debris, peristalsis, or suboptimal insufflation. The aforementioned limitations underscore the critical challenges that must be addressed before such models can be reliably deployed across diverse populations in real-world clinical settings.

#### 3.3.2. CADx: WLE Versus NBI Endoscopy for Diagnosis of EGC

Another pivotal systematic review and meta-analysis included video data with comparisons between ML models and different endoscopic imaging techniques. Minfang Lv et al. evaluated CADx in EGC with several key distinctions. The authors incorporated both static image and dynamic video data, systematically compared different AI model architectures, and examined performance across different endoscopic imaging techniques such as WLE and NBI [32]. The authors concluded that the AI models demonstrate excellent diagnostic accuracy overall, with deep convolutional neural networks (DCNNs) outperforming traditional CNNs. Additionally, the review suggests NBI modalities show superior sensitivity and specificity over non-NBI methods. Furthermore, AI models in dynamic video were able to achieve similar diagnostic performance to their image counterparts [32].

A total of 26 studies comprising 43,088 patients up to January 2025 were included with histopathology as the reference standard. Crucially, subgroup analyses were performed by AI model type, distinguishing DCNN from traditional CNN and non-DL models such as support vector machines. The review captured studies utilizing both static images and dynamic video footage across NBI and non-NBI modalities. Overall, AI models demonstrated a robust pooled sensitivity of 0.90 (95% CI: 0.87–0.93), specificity of 0.92 (95% CI: 0.87–0.95), and an area under the curve (AUC) of 0.96 (95% CI: 0.94–0.98). Subgroup analysis of ML model types revealed important performance differences. DCNNs achieved higher sensitivity than traditional CNNs (0.94 vs. 0.89), while specificity remained comparable (0.91 for both). Notably, in the subset of studies employing dynamic video verification, the AI models’ AUC reached 0.98, which exceeded the clinician performance range (AUC 0.85–0.90). However, this data subset was not published for full evaluation. Subgroup analysis comparing NBI over non-NBI modalities demonstrated clear diagnostic superiority (predominantly WLE, pooled sensitivity 0.94 vs. 0.85 and specificity 0.94 vs. 0.87). The performance gap was further reflected in the diagnostic odds ratio (243.00 vs. 39.73) and positive likelihood ratio (15.00 vs. 6.75), accompanied by a notably lower false-positive rate (6.3% vs. 12.6%). Most importantly, further analysis confirmed imaging modality as a statistically significant contributor to the observed heterogeneity (*p* = 0.018 for global comparison), underscoring NBI as the modality choice for superior AI diagnostic performance [32].

The superior accuracy associated with NBI likely stems from its intrinsic optical properties. By enhancing visualization of mucosal microvascular architecture and surface patterns, NBI provides richer feature sets for DL algorithms to exploit during classification tasks. This improved delineation of lesion boundaries and tissue characteristics facilitates more precise differentiation between neoplastic and non-neoplastic mucosa (6). From a clinical perspective, NBI examination is restricted mostly due to time constraints, leading to suboptimal compliance and subsequent reduction in quality. AI-assisted endoscopy allows clinicians to overcome this limitation. However, there have been few studies assessing the impact on clinical workflow, namely the impact on detection rates of other ‘off-target’ lesions. Although the study’s primary objective was not to conduct a head-to-head statistical comparison of AI performance between modalities, nonetheless the results highlight the importance of stratifying AI studies by imaging modality, as pooled estimates combining heterogeneous techniques may obscure meaningful performance differences relevant to clinical implementation decisions. Yet, there are limitations to generalizability in this pooled analysis. Importantly, there is heterogeneity in the utilization of endoscopic modalities across studies. For example, some may have used NBI only for suspicious lesions identified on WLE, which precludes a direct comparative analysis. Lastly, with only 11.5% of patient data from three prospective studies [32], the predominance of retrospective single-center designs also once again limits generalizability.

#### 3.3.3. CADx: Enhancing Diagnosis in Less Experience Endoscopist

A key strength in AI endoscopy is the ability to mitigate the EGC diagnostic capabilities of less experienced endoscopists. This systematic review and meta-analysis by Shi et al. illustrates the ability of ML models to elevate operators’ EGC diagnostic rate to a level comparable to specialists [33]. A total of 21 studies comprising 16,074 patients up to September 2022 were included with histopathology as the reference standard. Critically, separate pooled analyses were performed for specialist clinicians (*n* = 76), non-specialist clinicians (*n* = 72), and both groups with ML assistance. ML models demonstrated excellent standalone diagnostic performance. However, their greatest clinical value lies in augmenting human judgment, particularly for less experienced endoscopists, which is a significant portion of the workforce. The systematic review demonstrates that non-specialist clinicians’ sensitivity improved significantly from 0.64 to 0.76 (95% CI: 0.68–0.83) with ML support, bridging the diagnostic discrepancy to the level of unaided specialists [33].

A limitation pertinent to this study is the exclusion of poor-quality images within the dataset. This is particularly important as poor-quality images are more prevalent within the practice of less experienced endoscopists, potentially overestimating real-world performance. Additionally, while ML assistance improved non-specialist sensitivity, specificity did not show corresponding improvement, and specialist specificity paradoxically decreased slightly with ML assistance, suggesting that AI integration may introduce false positives and new diagnostic challenges.

#### 3.3.4. Real-World Data in Interventional Trials

While the aforementioned systematic reviews provide strong evidence of AI’s capability in controlled pre-clinical settings, prospective interventional data are essential to demonstrate real-world clinical benefit. In the current landscape, studies are limited and evidence remains conflicting.

A pioneering single-center RCT by Wu et al. demonstrated a reduction in gastric neoplasm miss-rate with the use of a DCNN WLE video-based system during live upper gastrointestinal endoscopy [30]. In this first-in-class tandem study, patients underwent an initial AI-assisted or routine endoscopic examination, immediately followed by the respective alternative, with biopsies performed after both procedures as indicated by AI and the endoscopist. In a cohort of over 1800 patients, the AI-assisted group demonstrated a significantly lower miss rate compared with the routine examination group (6.1% vs. 27.3%; relative risk 0.224; *p* = 0.015). The clinical significance of this finding is underscored by the substantial reduction in miss rates for smaller neoplasms (≤10 mm), which are notoriously difficult to detect during routine WLE. AI assistance significantly reduced the miss rate from 42.3% (95% CI 24.0–62.8; 11/26) to 9.4% (95% CI 2.5–26.2; 3/32, *p* = 0.0052) in this challenging subgroup [30]. Reassuringly, this improvement was achieved without a significant increase in examination time, supporting the practical feasibility of AI integration into clinical workflow. However, given the single-center design and the inherent limitations of the tandem methodology, where the same endoscopist performed both examinations, these promising results require validation in larger, multicenter settings.

Recently, Higuchi K et al. addressed this research gap with a multi-center study. Instead of employing a tandem comparison, the study used the expert’s findings as the diagnostic reference. However, the study failed to demonstrate diagnostic improvement of gastric neoplasms in non-expert endoscopists [31]. Over 300 patients were randomized to AI-assisted or routine endoscopic examination. In this study, under DCNN WLE image-based endoscopy, non-experts were tasked with completing the examination first, then examination by the expert endoscopists followed with or without AI assistance. NBI examination was allowed at the end of both examinations, at the expert endoscopists’ discretion. The experts’ findings served as the reference standard. More importantly, non-neoplastic lesions with suspicious endoscopic features but pathologically benign were included. This study design more accurately reflects real-world practice, where a significant portion of lesions are eventually proven to be non-neoplastic. Although the study failed to demonstrate statistically significant diagnostic improvement, the study provided some insights into the pattern of AI errors: the system demonstrated very high sensitivity but extremely low specificity (95.7% vs. 7.4%), indicating a strong bias towards overdiagnosis. Analysis of missed lesions revealed that flat or superficially depressed small neoplasms (≤9 mm) were more likely to be misclassified. While these findings offer some understanding of the misdiagnosis, a detailed analysis of the AI errors, such as in relation to histological subtypes or anatomical locations, was not reported.

A growing body of interventional evidence has now reached sufficient volume to support meta-analysis of RCTs alone. Al Hayek and colleagues have published the most comprehensive synthesis to date, comprising 11 trials and over 57,000 patients, evaluating AI-assisted endoscopic detection of all neoplastic subtypes encountered during upper gastrointestinal endoscopy [34]. The meta-analysis demonstrated that AI-assisted endoscopy significantly outperforms conventional examination in overall neoplasm detection (RR 1.57, 95% CI 1.23–2.01). In the stomach specifically, detection of high-grade intraepithelial neoplasia (HGIN; RR 1.75, 95% CI 1.44–2.11) and carcinoma (RR 1.66, 95% CI 1.37–2.02) showed consistent benefit with no observed heterogeneity (I^2^ = 0% for both). In contrast, while overall gastric neoplasm detection (RR 1.68, 95% CI 1.21–2.33) and low-grade intraepithelial neoplasia (LGIN; RR 1.74, 95% CI 1.09–2.78) also suggested strong potential, both were associated with substantial heterogeneity (I^2^ ≈ 60%). Crucially, this meta-analysis included only three studies specifically targeting the stomach, significantly limiting the generalizability and robustness of its gastric-specific conclusions.

The discordant findings between these RCTs highlight the substantial variability that currently exists, not only in AI model architecture and how such systems are deployed in clinical practice, but also in how research studies are designed. Future standardized methods are required to further ascertain the effectiveness of AI-assisted detection and diagnosis, ideally with incorporation of NBI to assess generalizability across different imaging modalities.

### 3.4. Beyond Diagnosis: AI Guidance for Endoscopic Therapy in EGC

Successful endoscopic curative resection is dependent on appropriate and reliable case selection. Key features such as cell differentiation type, depth of invasion, and lymphovascular invasion (LVI) confer risks of LNM and subsequent metastatic progression. Effective resection relies on selecting cases with the lowest risk. Most commonly, these are determined endoscopically with both WLE and NBI. Definitive local staging is only available after resection, where often a mismatch occurs between endoscopic and actual staging as determined by pathology [5]. AI models aim to mitigate the discrepancy, in hopes of guiding curative resection in EGC. In this section, we present the latest studies and systematic reviews on AI guidance in endoscopic resection of EGC.

#### 3.4.1. CADx of Critical Endoscopic Features for Curative Resection

A systematic review and meta-analysis by Agarwal et al. evaluated the performance of CNN-based models for predicting depth of invasion in EGC [35]. Ultimately, the review concluded AI has potential for predicting depth of invasion, illustrating robust accuracy, sensitivity and specificity. However, the authors also concluded the systematic review is significantly limited by the small number of studies included. Notably, sensitivity analysis after excluding one influential study reduced both pooled sensitivity and specificity to 78% (95% CI: 75–81%) [35], underscoring the fragility of the current evidence base. While these findings suggest some potential for AI-assisted characterization of EGC, the high degree of heterogeneity across a limited number of studies highlights a fundamental challenge. Even for a well-defined task such as predicting depth of invasion alone, the existing literature reveals considerable variability in model design, imaging protocols, and reference standards. This inconsistency limits the development of a robust and generalizable AI model for local staging and reinforces the need for larger, prospectively validated, and standardized datasets before such tools can be reliably integrated into clinical decision-making.

While single-feature prediction models may enhance specific aspects of endoscopic resection, the field is progressively shifting towards more ambitious objectives. Recent research has increasingly focused upon developing AI systems capable of predicting multiple critical pathologic outcomes simultaneously, thereby offering a more comprehensive assessment from a single-feature endoscopic evaluation. Lee et al. present a novel AI system capable of predicting multiple critical pathological outcomes from pre-operative WLE images and videos [36]. The model demonstrated high accuracy and significantly outperformed expert endoscopists in predicting differentiation status, LVI and LNM (overall accuracy 92.7% vs. 71.6% of expert endoscopists). However, when considering other parameters such as sensitivity, specificity, positive and negative predictive rates, the AI model was only more reliable and significantly favorable in predicting submucosal invasion (83.3% vs. 70.9% for submucosal invasion) [36]. This highlights the nuance between overall accuracy and the ability to correctly identify positive cases. Although the findings were promising, external validation with data from a separate institution revealed a notable decline in AI performance. The decline was emphasized in particular subtypes such as undifferentiated histology and submucosal invasion; however, it did not provide granular qualitative analysis of whether specific anatomical locations or lesion morphologies were more prone to misclassification.

Overall, this study by Lee et al. demonstrates the conceptual potential of a multi-task AI system capable of characterizing several key EGC features from a single endoscopic evaluation. However, the model’s sensitivity for critical pathological features such as LVI and LNM remained consistently poor, at best comparable to expert endoscopists [36]. This limitation hinders its ability to effectively reduce existing diagnostic discrepancies. The realization of a robust combined model capable of reliably determining whether an EGC is suitable for endoscopic resection, through simultaneously achieving high accuracy and sensitivity across all relevant histological parameters, would represent a significant advancement over the current paradigm. Such a tool remains an aspirational goal, contingent upon overcoming the persistent challenges of class imbalance and, crucially, the limitations of sub-optimal generalizability across diverse conditions, a need starkly underscored by the performance degradation observed during external validation.

#### 3.4.2. Predicting Lymph Node Metastasis for Non-Curative Resection

The limitations of endoscopic evidence-only models have prompted the exploration of more comprehensive approaches that integrate endoscopic findings with clinical and pathological data to improve prediction of LNM and guide post-resection management. The eCura system, a widely adopted risk-scoring tool based on post-operative pathological features, is currently used to stratify LNM risk after non-curative endoscopic submucosal dissection (ESD), helping clinicians decide whether additional surgery is warranted. However, its performance remains suboptimal, with limited discriminative ability in certain patient subgroups.

Addressing this gap, Mao et al. developed a DL-based decision-support system integrating endoscopic images with clinical data to predict LNM in EGC patients prior to ESD [37]. In a multi-center retrospective study of 605 patients, it demonstrated robust predictive performance for pre-ESD LNM, achieving AUC values ranging from 0.843 to 0.875 across external validation cohorts. Beyond its standalone accuracy, the model also proved its clinical utility by improving endoscopists’ diagnostic accuracy by 13.8%, driven primarily by a significant increase in specificity (mean increase of 0.17, *p* = 0.03) [37]. Practically, this improvement would theoretically translate to fewer unnecessary surgeries without missing true LNM cases.

Critically, the study also introduced a post-ESD model, incorporating pathological data from the resected specimen. The AI model significantly outperformed the eCura system (AUC of 0.91 vs. 0.71, *p* < 0.01). Most importantly, this theoretically translates into a clinically meaningful reduction in unnecessary additional surgeries by 24.2%, again achieved without any false negatives for LNM [37]. These findings illustrate a promising paradigm where multimodal AI systems, capable of integrating diverse data types, may eventually support the entire clinical pathway for EGC—from initial diagnosis and treatment selection to post-resection surveillance and risk stratification. By surpassing current risk stratification tools, such systems move closer to the goal of comprehensive, individualized patient management, addressing not only the diagnostic mismatch but also the therapeutic decision-making that follows. Its limitations in retrospective design and a single ethnic group are actively being addressed by the authors with future validation studies. This direction towards a multimodal AI system is particularly beneficial, as in routine clinical practice, endoscopists’ s decisions encompass a wealth of non-imaging data, ranging from demographics and relevant history to clinical data such as other non-endoscopic assessment modalities. The majority of current AI models encompass endoscopic image-only data. This gap highlights a key area for future AI development: the integration of multimodal data to better emulate and augment the comprehensive diagnostic process of expert clinicians.

#### 3.4.3. Delineation of Endoscopic Resection Margins

Beyond the identification of lesions suitable for endoscopic resection, recent advances have demonstrated the potential of AI to guide treatment through the delineation of resection margins. Ling et al. developed and validated a CNN-based system capable of simultaneously identifying the pathological differentiation status and delineating the margins of EGC on magnifying NBI, achieving superior performance compared with expert endoscopists and demonstrating real-time functionality in selected clinical video footage [38]. For predicting differentiation status, the authors employed two mature deep CNN architectures pretrained on ImageNet: VGG-16 and ResNet-50, with the former ultimately selected for further study due to its superior accuracy. For delineating the extent of EGC, they implemented UNet++, a novel and powerful architecture specifically designed for medical image segmentation. The retrospective study was conducted across two Chinese centers and utilized ME-NBIs from patients with pathologically confirmed EGC following endoscopic resection. The findings remained consistent through external validation. Additionally, the system successfully delineated margins on frozen frames during real-time video testing on two real magnifying-NBI videos, demonstrating potential feasibility. However, this demonstration was largely qualitative, as quantitative evaluation remained confined to still images. While Ling et al. illustrate the promise of CNN-based systems for treatment guidance, several factors limit their generalizability to the actual conduct of endoscopic resection. Such procedures demand real-time margin delineation during dynamic video examination, often under suboptimal imaging conditions complicated by bleeding, mucus, or the presence of staining dyes. Moreover, EGC resection is inherently a dynamic process, during which submucosal injection and mechanical manipulation alter mucosal morphology and lesion conformation. Future AI models capable of maintaining accurate delineation despite these intra-procedural challenges would better serve their intended clinical purpose.

### 3.5. Obstacles and Limitations to AI Endoscopy in EGC

The studies reviewed attest to the considerable promise of AI in enhancing the endoscopic diagnosis and treatment of EGC. As with all data-driven technologies, continued advances in model architecture and training paradigms will inevitably yield incremental improvements in accuracy and related performance metrics. Nevertheless, several fundamental challenges that impede clinical translation remain. These include well-recognized technical obstacles throughout all clinical domains of AI, such as class imbalance arising from low event prevalence, significant heterogeneity in study methodology and reporting standards [39], and inherent disparities related to hospital and equipment compatibility [40]. More specifically, AI research in EGC faces unique constraints. The lack of objective performance metrics, in contrast to adenoma detection rate (ADR) that serves as a benchmark in colonoscopy, combined with the absence of universally accepted standards for robust EGC-related studies, represents a critical barrier to progress. These issues, which we will discuss in this section, fundamentally hinder generalizability and implementation in routine clinical practice.

The absence of a validated benchmark for EGC detection stems from several interconnected factors. Foremost is the intrinsically low event rate of gastric neoplasia in screening populations. A recent meta-analysis found a pooled gastric cancer detection rate of only 0.76%, with EGC constituting just over half of detected cases [41]. In contrast, adenoma prevalence in colonoscopy exceeds 3% in average-risk populations, which enables statistically robust comparisons of performance between endoscopists [42]. The nature of ADR works precisely because the underlying event is sufficiently common to generate meaningful performance variation. Additionally, unlike the adenoma-carcinoma sequence in the colon, the gastric carcinogenesis cascade lacks a direct precursor lesion with linear, predictable progression to cancer. A recent meta-analysis demonstrated that progression rates vary substantially by geographical risk, with annual incidence per 1000 person-years in low versus high gastric cancer incidence countries of 0.97 versus 2.47 for atrophic gastritis, 2.37 versus 3.47 for intestinal metaplasia, and 5.51 versus 14.80 for dysplasia [43]. More importantly, ADR is strongly correlated with post-colonoscopy cancer incidence [44], no comparable measure exists for the stomach. The most formidable obstacle lies in the pathological discrepancies within gastric precursor lesions. Illustrated by Dong et al., a recent multi-center RCT of AI for gastric neoplasm detection, centralized expert pathology review resulted in the downgrading of 83.58% (804 of 962) of LGIN and 14.00% (7 of 50) of HGIN diagnoses to benign lesions [29]. This degree of diagnostic instability would render any performance metric built upon local pathology fundamentally unreliable, and the infrastructure required for centralized review is impractical for routine quality monitoring. These fundamental constraints suggest AI in EGC will require a different validation paradigm. Perhaps combining process measures with rigorous centralized outcome adjudication, rather than seeking a singular metric analogous to ADR. The absence of a validated benchmark, however, represents only one facet of a broader challenge.

The field has yet to reach consensus on fundamental requirements for robust EGC studies, including minimum dataset composition requirements, appropriate validation strategies, and clinically meaningful endpoints. While initiatives such as the Quality Assessment of pre-clinical AI studies in Diagnostic Endoscopy (QUAIDE) consensus have proposed standardized evaluation frameworks for AI in diagnostic endoscopy [45], widespread adoption and enforcement of these standards remain aspirational. Until the field can establish consensus, the translation of promising AI models from research publications to impactful clinical tools will remain constrained.

#### Current Research Design Paradigms and Limitations

Despite the challenges outlined above, several landmark studies have begun to tackle key limitations through rigorous trial design.

The foundational work by Wu et al., in a single-center tandem RCT, directly addressed the absence of a validated benchmark for assessing AI performance in EGC diagnosis [30]. This methodology, however, is not without its inherent practical constraints and potential biases. The tandem design requires patients to undergo two consecutive procedures, increasing procedural time, resource utilization, and patient burden. More fundamentally, the validity of the second examination as a true reference standard is susceptible to the “informed second look” phenomenon. Where endoscopists are aware of performing a verification exam, they may exercise greater vigilance than in routine practice. This potentially inflates the apparent miss rate of the initial procedure. Additionally, the “vanishing lesion” problem poses a particular challenge, as subtle lesions may be difficult to re-identify during the second pass, leading to potential misclassification of missed lesions.

In contrast, Dong et al. adopted a parallel design to investigate the real-world effectiveness of AI-assisted EGC detection [29]. In this design, patients are randomized to receive either AI-assisted or routine endoscopy. While this approach avoids the behavioral biases inherent to tandem designs, it demands a substantially larger sample size to achieve adequate statistical power, owing to increased inter-patient variability. Furthermore, the logistical and resource challenges associated with a multicenter parallel trial are amplified by the requirement for such a large cohort. Notably, this 24-center trial of nearly 30,000 patients incorporated a prespecified centralized expert endoscopist and pathologist review as the cornerstone of its primary outcome analysis. By subjecting all detected suspicious lesions to blinded review by expert panels, the study established a robust “ground truth” diagnosis. As mentioned previously, a significant proportion of LGIN and HGIN lesions were downgraded to benign lesions. Critically, it showed that the apparent benefit of AI-assisted diagnosis based on local pathology (4.06% vs. 3.57%, *p* = 0.03) disappeared when measured against this centralized gold standard (1.42% vs. 1.25%, *p* = 0.25). This finding does not diminish AI’s potential but fundamentally redefines the validation paradigm, demonstrating rigorous endpoint adjudication is not merely an adjunct, but an absolute prerequisite for credible AI trials in EGC.

Each study design carries inherent strengths and limitations. Tandem designs, where the same patient serves as their own control, eliminate inter-patient variability and provide a direct, highly sensitive measure of AI’s capacity to detect lesions that would otherwise be missed. Conversely, parallel designs, where patients receive only one examination, mirror real-world clinical practice and capture the AI’s overall net clinical benefit, including operator variability and workflow integration. Future iterations of clinical evaluation should integrate the complementary advantages of both designs to overcome the fundamental challenges posed by EGC, ideally in conjunction with centralized outcome adjudication.

## 4. Discussion

**Figure 5:**
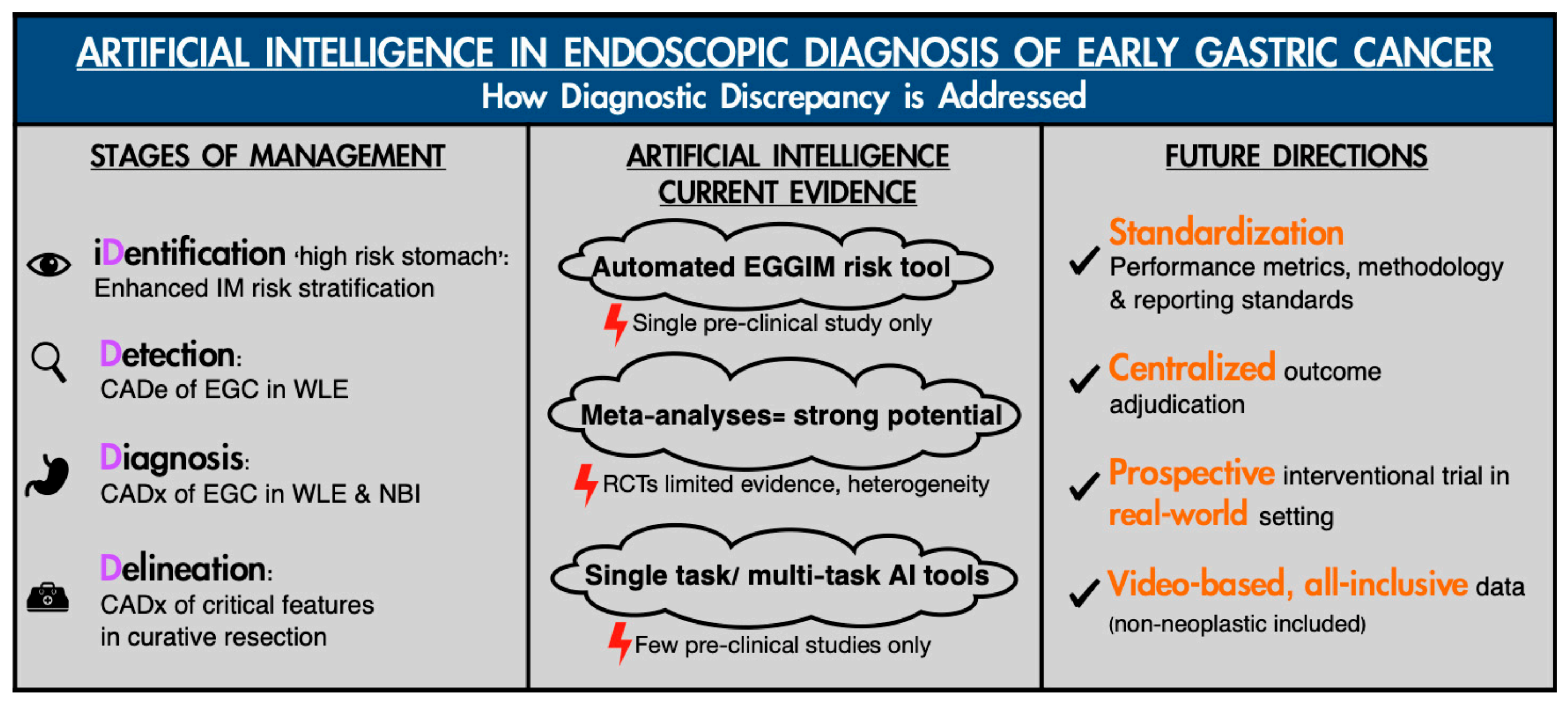
The impact of artificial intelligence on the endoscopic diagnosis of EGC. Summarized with each key step of the clinical pathway and the state of the latest evidence highlighted.

Mismatch between endoscopic and pathological diagnosis remains a persistent and clinically significant challenge across the spectrum of gastric neoplasm. These inconsistencies frequently lead to substantial alterations in patient management. As this review has demonstrated, AI-assisted diagnostic tools possess the potential to mitigate these differences by augmenting endoscopic interpretation at every stage of the clinical pathway (Figure 5). Systems capable of automated EGGIM scoring can identify high-risk populations with high sensitivity, overcoming inter-observer variability in recognizing subtle mucosal patterns. DL models applied to WLE and NBI achieve diagnostic accuracy comparable to expert endoscopists, with particular benefit for less experienced practitioners, for whom variability commonly compromises patient care. Furthermore, emerging multi-task models capable of simultaneously predicting critical pathological features, risk-stratifying post-resection, and even delineating resection margins in real time, illustrate a trajectory toward comprehensive AI guidance that extends beyond diagnosis to directly guide therapeutic decision-making.

Nevertheless, the trajectory from promising research to routine clinical implementation remains obstructed by fundamental challenges that cannot be overcome by algorithmic improvements alone. The absence of validated objective performance metrics analogous to ADR in colonoscopy, the intrinsically low event rate of gastric neoplasia, and the profound pathological discrepancies in precursor lesion diagnosis collectively hinder the development of robust, generalizable models. Performance degradation between internal and external validation remains the rule rather than the exception, reflecting the field’s reliance on retrospective, single-center datasets and curated still images that poorly approximate real-world clinical conditions. While initiatives such as the QUAIDE consensus provide valuable frameworks for standardized evaluation, widespread adoption remains aspirational. Addressing these limitations will require a concerted shift toward prospective, multicenter trials with centrally adjudicated outcome review, real-time video validation, and datasets representative of diverse populations and equipment. Only through such rigorous methodology can AI-assisted endoscopy transcend its current constraints and resolve the discrepancy. Although the latest RCTs are trending towards a more effective direction, interventional evidence remains conflicting and heterogeneous in design. Ultimately, the goal of AI systems should be beyond matching human performance but reliably overcoming the mismatches between endoscopists and between endoscopic interpretation and pathological truth, which continue to compromise outcomes for patients with EGC.

Looking ahead, several priorities emerge to guide the field toward meaningful clinical impact. First, the field must embrace standardized reporting frameworks such as QUAIDE to ensure methodological transparency and reproducibility. Second, future AI systems should prioritize real-time video analysis over still image classification, with prospective validation in diverse, real-world clinical settings encompassing varying equipment, populations, and operator expertise. Third, the development of multi-task models capable of simultaneously predicting differentiation status, depth of invasion, LVI, and LNM, whilst maintaining acceptable sensitivity across all parameters represents the ultimate aspirational goal. Such a tool would not only identify appropriate candidates for endoscopic resection but also guide the procedure itself through accurate margin delineation, even under the challenging conditions of intra-procedural bleeding and tissue manipulation. Achieving this vision will require not merely larger datasets, but smarter data: prospectively collected, centrally adjudicated, and representative of the full spectrum of gastric pathology and clinical scenarios. Once sufficient evidence demonstrates robust generalizability across diverse clinical scenarios; however, the most formidable challenges will shift from technical development to clinical implementation. Fundamental questions regarding the optimal design of management pathways, specifically what role AI should assume within clinical workflows, remain largely unresolved. The prevailing consensus favors a ‘human-in-the-loop’ framework, wherein AI augments rather than replaces clinical judgment. This aims to serve as a triage and decision-support tool, while the endoscopist retains ultimate accountability. Yet, even this model raises complex liability questions: who bears responsibility when AI-driven recommendations prove erroneous? Current legal and ethical frameworks provide few clear answers, exposing clinicians to uncertainty and threatening trust in doctor-patient relationships. Regulatory hurdles compound these challenges. The ‘black-box’ nature of DL systems continues to impede explainability and clinical acceptance, while the paucity of AI systems that have successfully navigated regulatory approval—despite the existence of dedicated Software As A Medical Device frameworks in jurisdictions such as the US and Japan—underscores the rigor of the pathway ahead. Moreover, the emergence of generative AI introduces novel risks, including the potential for ‘hallucinations’ that may evade even stringent regulatory oversight. Addressing these translational and governance challenges will be as critical to the future of AI in ECG as achieving high diagnostic accuracy. Until then, the revolution in AI-assisted endoscopy for EGC remains a distant future.

## 5. Conclusions

The discrepancy between endoscopic and pathological diagnoses remains prevalent throughout the clinical management of EGC. AI is positioned to overcome current diagnostic shortcomings. However, fundamental limitations hinder effective and robust assessment. This aspirational goal currently remains unaddressed. Future standardized research is crucial prior to consideration of widespread implementation.

## Figures and Tables

**Table 1:** Summary of the latest clinical trials on AI-assisted endoscopic quality assurance, detection and diagnosis of early gastric cancer.

**Computer-Aided Quality Assessment (CAQ)**								
**First Author [Ref] (Year)**	**Mode of** **Examination**	**AI Model**	**Endoscopist Experience Level**	**Center** **Number (s)** **and** **Country**	**Total** **Number of** **Lesions** **/Patients**	**Quality Control** **Metrics**	**AI Assistance**	**Control**
Zhou et al. [24] (2024) ^	WLE	Intelligent Quality Control System	12 inexperienced 12 experienced	6, China	48 lesions	Mean number of blind spots = Percentage of acceptable visibility after cleaning = (%) Mean inspection time = (minutes)	2.3 (SD 2.5) 98.9–100% 9.6 (SD 5.23)	6.2 (SD 3.7) * 87.9–88.9% * 9.6 (SD 5.04)
Chen et al. [21] (2025) ^	WLE	Endoscopy Quality Control Assistant	All endoscopists with 3–5 years of experience	7, China	1658 lesions	Mean operation score = (range) Mean inspection time = (minutes [range]) Biopsy rate = (% [range])	86.37 (81.13–93.90) 6.84 [6.28–7.23] 88.83 [83.1–92.8]	73.64 (65.76–89.15) * 5.6 [4.99–6.82] * 77.9 [70.7–86.3] *
Dong et al. [29] (2026) ^	WLE	ENDOANGEL-GN	17 junior 30 senior 51 expert	24, China	26,001 patients	Mean number of blind spots = Mean procedure time = (minutes) Mean inspection time = (minutes) Biopsy rate % = (ITT) [PP]	1.07 7.69 7.35 53.75 [53.69]	2.52 * 7.33 * 6.99 53.01 [52.15] *
**Computer Aided Detection (CADe)**								
**First Author [ref] (year)**	**Mode of** **Examination**	**AI Model**	**Endoscopist** **Experience Level**	**Centre Number (s) and Country**	**Total Number of** **Lesions/patients**	**Detection Metrics**	**AI Assistance**	**Control**
Wu et al. [30] (2021)	WLE	ENDOANGEL-LD	2 junior 4 senior	1, China	93 lesions	Detection Rate, All gastric neoplasm = (%) [RR and CI] LGIN = (%) HGIN = (%) Carcinoma = (%) Miss Rate of all gastric neoplasms = (%) [RR and CI]	4.9 RR 1.372 CI 0.88–2.14 2.5 0.1 2.1 6.1 RR 0.224 CI 0.07–0.74 *	3.5 1.8 0.2 1.5 27.3
Zhou et al. [24] (2024) ^	WLE	Intelligent Quality Control System	12 non-experienced 12 experienced	6, China	48 lesions	Detection Rate, All early upper GI neoplasm = (%,incl. non-gastric) [RR and CI] LGIN = (%) [RR and CI] HGIN = (%) [RR and CI] Carcinoma = (%)	6.1 RR 2.670 CI 1.59–4.52 * 2.4 RR 3.671 CI 1.42–10.07 * 1.4 RR 3.254 CI 0.99–11.78 * 0.3	2.3 0.7 0.4 0.0
Chen et al. [21] (2025) ^	WLE	Endoscopy Quality Control Assistant	All endoscopists with 3–5 years of experience	7, China	1658 lesions	Detection Rate, All early upper GI neoplasm = (incl. non-gastric, %) [RR and CI] LGIN = (%) [RR and CI] HGIN = (%) [RR and CI] Carcinoma = (%) [RR and CI]	8 RR 1.442 CI1.33–1.57 * 3.04 RR 1.380 CI 1.21–1.58 * 1.71 RR 1.380 CI 1.26–1.58 * 1.51 RR 11.680 CI 1.37–2.06 *	5.55 2.20 1.00 0.9
Dong et al. [29] (2026) ^	WLE	ENDOANGEL-GN	17 junior 30 senior 51 expert	24, China	26,001 patients	Detection Rate, Gastric neoplasm With central review = (%) [RR and CI] Gastric neoplasm Without central review = (%) [RR and CI]	1.42 RR 1.13 CI 0.92–1.38 4.06 RR 1.14 Cl 1.14–1.28 *	1.25 3.57
**Computer-Aided Diagnosis (CADx)**								
**First Author [ref] (year)**	**Mode of** **Examination**	**AI Model**	**Endoscopist Experience Level**	**Centre Number (s) and Country**	**Total Number of Lesions /patients**	**Performance Metrics**	**AI Assisted**	**Control**
Higuchi et al. [31] (2026)	WLE	gastroAI-model β	Non-expert	429 lesions	8, Japan	Non-experts diagnostic accuracy for gastric neoplasia against pathology Accuracy (%) = Sensitivity (%) = Specificity (%) = Positive Predictive Value (%) = Negative Predictive Value (%) =	65.3 68.6 60.8 72.1 56.6	59.9 63.9 53.3 70.9 45.5

CAQ: Computer Aided Quality Assessment; CADx: Computer Aided Diagnosis; CADe: Computer Aided Detection; WLE: White light examination; LGIN: Low-grade intraepithelial neoplasia; HGIN: High-grade intraepithelial neoplasia; ITT: Intention to treat analysis; PP: Per protocol analysis; RR: Relative risk; CI: 95% Confidence Intervals; Operation score: composite measure derived from a three-step calculation that evaluates the examination quality at the level of individual video frames, specific anatomical parts, and the entire upper gastrointestinal (UGI) tract; * Denotes statistical significance of at least *p* ≤ 0.05; ^ Denotes AI model with CAQ and CADe functions assessed in same study. From clinical trials included in this narrative review literature search.

## Data Availability

No new data were created or analyzed in this study. Figure 1 demonstrates early gastric cancer in different modalities to illustrate the process of endoscopic diagnosis including AI-assisted detection with OIP-Ge1 (Olympus Corporation, Tokyo, Japan). The image is captured by the authors. Figure 3 demonstrates the application of multi-functional artificial intelligence platform in endoscopic diagnosis of early gastric cancer in NBI examination, with EndoAngel (EndoAngel UGI, Wuhan, China). The image is captured by the authors. Figure 4 demonstrates the application of endoscopic image diagnosis support software in white light examination of early gastric cancer, with gastroAI-model G (AI Medical Service Inc, Japan). The image is captured by the authors. Data sharing is not applicable to this article.

## Abbreviations

The following abbreviations are used in this manuscript:

AI	Artificial intelligence
ADR	Adenoma detection rate
CADe	Computer Aided Detection
CADx	Computer Aided Diagnosis
CAQ	Computer Aided Quality Assessment
CNN	Convolutional Neural Network
DCNN	Deep convolutional neural networks
DL	Deep learning
EGC	Early gastric cancer
EGGIM	Endoscopic Grading of Gastric Intestinal Metaplasia
ESD	Endoscopic submucosal dissection
GIM	Gastric intestinal metaplasia
HGIN	High-grade intraepithelial neoplasia
LGIN	Low-grade intraepithelial neoplasia
LNM	Lymph node metastasis
LVI	Lympho-vascular invasion
ML	Machine learning
NBI	Narrow band imaging
RCT	Randomized Control Trial
WLE	White light endoscopy
QUAIDE	Quality Assessment of pre-clinical AI studies in Diagnostic Endoscopy
